# The ferroptosis-related long non-coding RNAs signature predicts biochemical recurrence and immune cell infiltration in prostate cancer

**DOI:** 10.1186/s12885-022-09876-8

**Published:** 2022-07-18

**Authors:** Chunhui Liu, Yue Gao, Jiaxuan Ni, Saisai Chen, Qiang Hu, Can Wang, Mingjin Hu, Ming Chen

**Affiliations:** 1 Department of Urology, Affiliated Zhongda Hospital of Southeast University, Nanjing, 210009 Jiangsu China; 2 Surgical Research Center, Institute of Urology, Medical School of Southeast University, Nanjing, 210009 Jiangsu China; 3 Department of Urology, Lishui People’s Hospital, Nanjing, 210009 Jiangsu China

**Keywords:** Ferroptosis, lncRNA, Immune cell infiltration, Prostate cancer, Prognostic model

## Abstract

**Background:**

Findings from numerous studies have revealed that ferroptosis is closely related to tumorigenesis and immune cell infiltration. Long non-coding RNAs (lncRNAs) are reportedly involved in the progression of various cancers, including prostate cancer (PCa). This study was designed to establish a ferroptosis-related lncRNA (frlncRNA) signature to predict PCa prognosis.

**Methods:**

The frlncRNAs were identified by studying their expression by Pearson’s correlation analysis. Differentially expressed prognosis related frlncRNAs were identified by the Wilcoxon test and univariate Cox regression analysis. The LASSO Cox regression model was used to build a model to predict biochemical recurrence (BCR) based on frlncRNAs. The GSEA software (version 4.1.0) was used to explore the enriched pathways in high- and low- risk groups. Patients with PCa were clustered into different subgroups by unsupervised clustering based on the frlncRNAs considered in the prognostic model. Real-time PCR and CCK8 assays were performed to verify the expression and function of frlncRNAs.

**Results:**

We identified 35 differentially expressed prognosis related frlncRNAs based on data on PCa from TCGA. A risk signature based on five frlncRNAs (AP006284.1, AC132938.1, BCRP3, AL360181.4 and AL135999.1), was confirmed to perform well in predicting BCR. The high-risk group had higher disease grades and a greater number of infiltrating immune cells. Besides this, we found that the five frlncRNAs were connected with typical immune checkpoints. With respect to molecular mechanisms, several metabolic pathways were found to enriched in the low-risk group. Furthermore, patients could be classified into different subtypes with different PSA-free times using the five frlncRNAs. Notably, AP006284.1, AC132938.1, BCRP3 and AL135999.1 were upregulated in PCa cells and tissues, whereas AL360181.4 exhibited the opposite trend. The downregulation of BCRP3 and AP006284.1 impaired the proliferation of 22RV1 cells.

**Conclusion:**

We generated a prognostic model based on five frlncRNAs, with clinical usefulness, and thus provided a novel strategy for predicting the BCR of patients with PCa.

**Supplementary Information:**

The online version contains supplementary material available at 10.1186/s12885-022-09876-8.

## Introduction

Prostate cancer (PCa) remains a common cancer type in men worldwide and accounted for 14.1% of total cancer cases in 2020 [1]. Radical prostatectomy and androgen deprivation therapy are standard strategies for PCa treatment [2]. In recent years, immunotherapy has brought major reforms to PCa therapy. Immunotherapy is acknowledged as an attractive treatment for malignancies and is considered a potentially useful method to improve the prognosis of individuals with tumors [3]. Typical immune checkpoints, such as PD-1, PD-L1, CTLA-4 and LAG-3, can exert immunomodulatory effects on the tumor microenvironment (TME) in PCa [4]. However, even though these treatments can control the disease in the long term, some patients continue to experience biochemical relapse (BCR) during follow-up. Therefore, it is of paramount significance to explore effective biomarkers for patients with PCa.

Ferroptosis is a newly discovered form of regulated iron-dependent cell death [5]. Ferroptosis is accompanied by the intracellular dysregulation of reactive oxygen species and the inactivation of GPX4, the core regulatory enzyme of the antioxidant system [6]. It has been confirmed to induce diverse physiological conditions [7], contributing to the progression of many diseases, such as Parkinson’s disease [8], and malignant tumors, including PCa [9–11]. Over the past 5 years, the interest in the role of ferroptosis in the development of PCa has increased in basic and clinical research. PCa cells respond to iron, whose combination with androgen receptor antagonists can aggravate oxidative damage, subsequently triggering cell death [12]. Oxidized lipids in ferroptotic cells can regulate the phagocytosis of macrophages to enhance immunity. However, the effect of ferroptosis on the prognosis of PCa has rarely been reported.

Long non-coding RNAs (lncRNAs) contain more than 200 nucleotides and cannot encode proteins [13]. They play important roles in several biological activities, including but not limited to, epigenetic regulation, cell cycle, and cell differentiation [14–16]. Owing to its distinct expression patterns, lncRNAs have garnered interest in research on different types of cancers. The lncRNA CCAT1 is considered to be an oncogene that accelerates the growth of PCa xenografts, leading to high mortality [17]. Evidence reported to data suggests that lncRNAs can regulate ferroptosis in cancer biology. For example, the lncRNA OIP5-AS1 regulates the miR-128-3p/SLC7A11 axis to suppress ferroptosis in PCa cells [18]. However, ferroptosis-lncRNA combinations are yet to be used in the prediction of PCa prognosis.

Here, we screened five frlncRNAs in PCa to construct a prognostic model that could be used for prognosis prediction and patient selection for immunotherapies.

## Methods

### Data collection

RNA-seq FPKM (fragments per kilobase million) data, somatic mutation data, and the corresponding clinical characteristics were obtained by searching UCSC Xena (https://xenabrowser.net/datapages/). To select lncRNAs, the annotation file of lncRNAs was extracted in the GENCODE databank (https://www.gencodegenes.org/). Data on clinical characteristics including age, race, Gleason score, TNM stage, PSA-free time, and PSA-free states, were extracted. A total of 495 patients with complete PSA-free follow-up information were included in the subsequent analysis. A total of 174 ferroptosis drivers and suppressors were identified from the FerrDb database (http://www.zhounan.org/ferrdb/index.html).

### Exploration of frlncRNAs

FrlncRNAs were identified by correlation analysis of expression data. First, the expression profiles of lncRNAs and ferroptosis-related genes in PCa were extracted by screening the TCGA database. Second, frlncRNAs were selected using Pearson’s correlation analysis with the absolute correlation coefficient > 0.30 and *P* < 0.001.

### Construction and validation of a prognostic gene model based on frlncRNAs

Differentially expressed frlncRNAs between PCa tissues and normal controls were identified using the limma package via the Wilcoxon test, with *P* < 0.05 as the threshold. The frlncRNAs with significant prognostic roles were selected by univariate Cox regression analysis, with *P* < 0.05 as the threshold. Next, patients were randomly divided into training groups (*n* = 330) and verification groups (*n* = 165). Following this, LASSO regression analysis was performed in the training group to develop a prognosis model using the “glmnet” R package. The selected frlncRNAs were used to develop a risk score. Based on the risk score the training and verification groups were divided into two groups. The predictive power was assessed using “survival” R and “survivalROC” R package. Principal component analysis (PCA) was performed using the “stats” R package. T-distributed stochastic neighbor embedding (t-SNE) was implemented using the “Rtsne” package.

### Independent prognostic analysis

We conducted univariate and multivariate Cox regression analyses to evaluate the independence of the model with traditional clinical characteristics in predicting the PSA-free survival of patients with PCa.

### Gene set enrichment analysis (GSEA)

GSEA was performed for the high-risk and low-risk groups according to the prognostic model using the GSEA software (version 4.1.0). The reference files of c2.cp.kegg.v7.4.symbols.gmt were downloaded from the Molecular Signatures Database (http://www.gsea-msigdb.org/gsea/msigdb/index.jsp). The statistical significance was set at FDR < 0.25 and *P* < 0.05.

### Immune cell infiltration analysis

Tumor purity data of PCa were retrieved from the ESTIMATE database (https://bioinformatics.mdanderson.org/estimate/index.html). The correlations of prognostic model gene expression with the scores of PCa were depicted as scatter plots. Next, immune cell infiltration was analyzed using CIBERSORT. Perl language was used to calculate the tumor mutation burden (TMB) of PCa. The correlations of prognostic model gene expression with TMB and microsatellite instability (MSI) were investigated by Spearman’s correlation analysis. The results were visualized using the fmsb package.

### Identification of subgroups of the PCa sample by consensus clustering

The 495 PCa samples were clustered into different subgroups by unsupervised clustering using the “ConsensusClusterPlus” R package. Clustering was applied by the k-means method with iterations of 50 and resample rate of 0.8. The optimal k value, representing the clustering number, was determined by the cumulative distribution function. The result was confirmed by PCA.

### Cell culture

Cell lines RWPE-1, PC3, Du145, LNCaP and 22RV1 were purchased from ATCC. Cells were cultured using RPMI-1640 medium (GiBCO, Thermo Fisher Scientific, USA) containing 10% fetal bovine serum. The incubator was set at 37ºC and 5% CO_2_.

### Quantitative real-time PCR (qRT-PCR)

The RNAeasy™ Animal RNAIsolation Kit with Spin Column (Beyotime Biotechnology, China) was used for total RNA extraction from cells and tissues. RNA was reverse transcribed to cDNA using the Servicebio RT First Strand cDNA Synthesis Kit (Servicebio, China). SYBR Green (Servicebio, China) was applied for real-time PCR analysis. Data were standardized to the expression of GAPDH. Primers were synthesized by SprinGen Biotech (China). PCR primers sequences were shown in Supplemental Table 1. The purity of RNA was evaluated based on an A260- A280 ratio of 1.8–2.0. The PCRs were repeated three times to validate the results.

### CCK8 assay

A proliferation assay was conducted using cell counting kit-8 (Biosharp, China). Two thousand cells transfected with si-BCRP3, si- AP006284.1 or si-NC were plated into each well of a 96-well plate. The absorbance in each well was measured at 0 h, 24 h, 48 h, 72 h and 96 h.

### Statistical analysis

R was used for statistical analysis in this study. The Wilcoxon test or Student’s *t*-test was used to compare differences in gene expression between groups. The relationships among gene expression, immune cell infiltration, TMB, MSI, and immune checkpoint gene expression were evaluated using Spearman’s or Pearson’s correlation coefficients. A *P* value < 0.05 was considered statistically significant.

## Results

### Development of the lncRNA signature

**Fig. 1:**
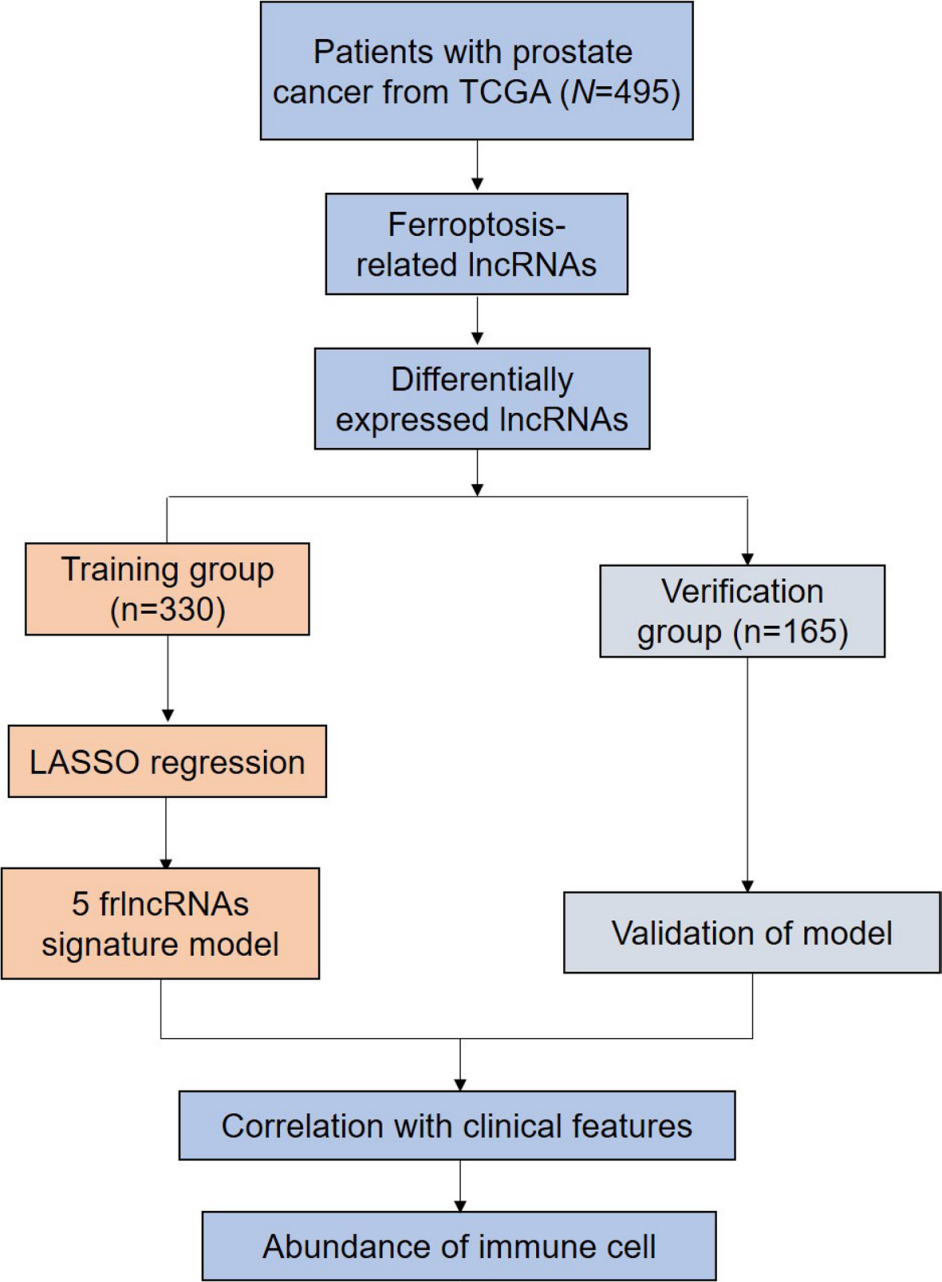
The working flow chart of this study

**Fig. 2:**
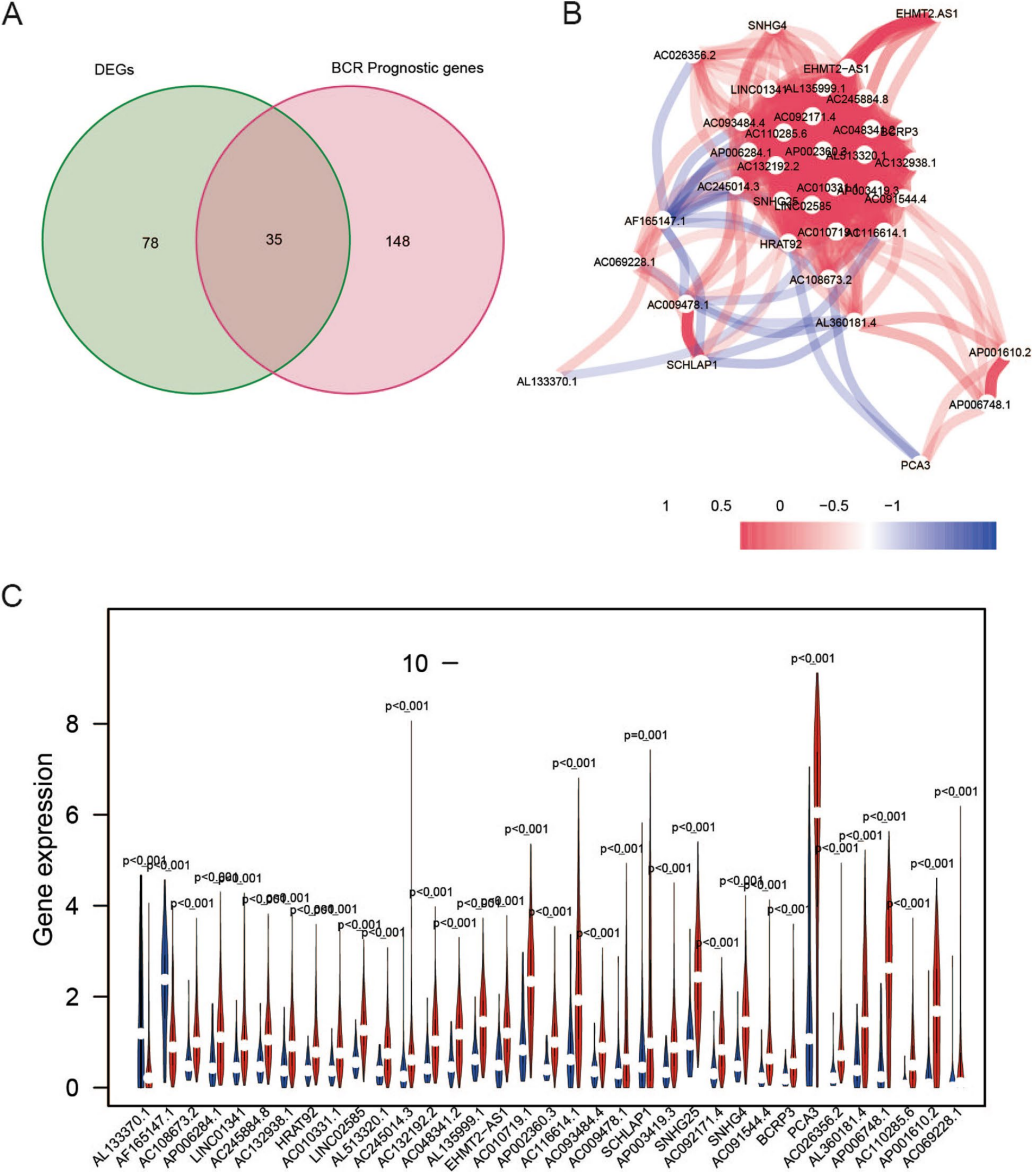
Screening of prognostic frlncRNAs. **A** The overlap of differentially expressed genes and BCR-related lncRNAs. **B** The interactive network of the 35 lncRNAs. **C** Violin plots showed the expression profiles of the 35 lncRNAs in PCa samples and normal controls

The schematic representation of the process is shown in Fig. 1. In total, 669 frlncRNAs were identified by Pearson’s correlation analysis (absolute correlation coefficient > 0.30 and *P* < 0.001) in 495 patients with PCa (Supplemental Table 2). Among the 669 lncRNAs, 183 were confirmed to be associated with BCR by univariate Cox analysis, with *P* < 0.05 as the threshold (Supplemental Table 3), and 113 lncRNAs were found to be differentially expressed between PCa and adjacent non-tumorous tissues. Thirty-five lncRNAs were identified on the overlap of data for 113 differentially expressed genes and 183 BCR-related genes (Fig. 2A). The interactive network demonstrated a strong correlation among the 35 lncRNAs (Fig. 2B). The relative expression of the lncRNAs was observed in PCa tissues and normal samples (Fig. 2C and Supplemental Fig. 1).

### Establishment of risk model based on frlncRNAs

**Fig. 3:**
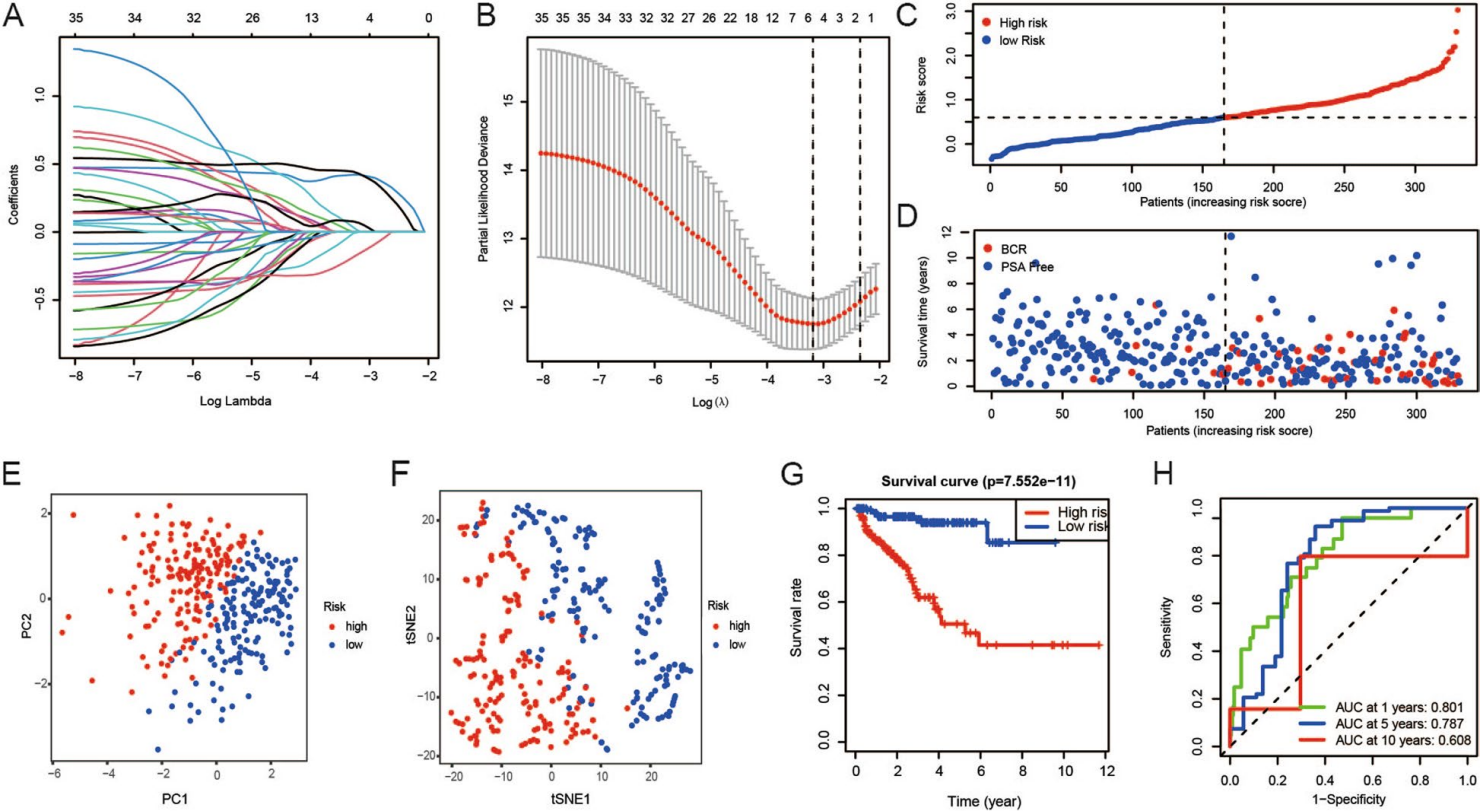
Construction of a prognostic model in the training group. **A**-**B** The risk score system was established using the LASSO Cox regression model. **C**-**D** The risk scores and BCR states in the training group. **E**–**F** PCA plot and t-SNE analysis of the training cohort. **G** KM plots suggested that patients with high risk scores displayed a markedly shorter PSA-free survival than those with low risk scores. **H** ROC showed the predictive value of the risk score for BCR

First, patients were randomly divided into the training group (*n* = 330) and the verification group (*n* = 165). The parameters of patients with PCa are shown in Supplemental Table 4. Following this, the relationship between the expression of frlncRNAs and PCa prognosis was confirmed by LASSO regression in 330 patients with PSA-free survival time and states in TCGA. Based on LASSO regression with minimum λ, AP006284.1, AC132938.1, BCRP3, AL360181.4 and AL135999.1 were selected to construct the model (Fig. 3A-B).

The risk score for each patient was computed. The following formula was developed based on the five frlncRNAs: risk score = 0.422 × AP006284.1 + 0.007 × AC132938.1 + 0.430 × BCRP3-0.160 × AL360181.4 + 0.068 × AL135999.1. The patients in the training group were divided into low-risk (165 patients) and high-risk categories (165 patients) based on the median risk scores (Fig. 3C-D). Patients with PCa in the high- and low-risk groups were categorized in two directions based on the findings from PCA and t-SNE analysis (Fig. 3E-F). The survival curves revealed that the patients in the high-risk group had a shorter PSA-free survival time (Fig. 3G). Time-dependent ROC analysis showed that this model was a good indicator for BCR, and the AUC was 0.801 for 1 year, 0.787 for 5 years, and 0.608 for 10 years (Fig. 3H).

### Neighbor gene network and interaction analysis of frlncRNAs

We conducted a comprehensive analysis of a network of the five frlncRNAs to explore their potential interactions with mRNAs in PCa. As expected, the expression of 53 genes was found to correlate with that of frlncRNAs (Supplemental Fig. 2A). Besides, AP006284.1, AC132938.1, BCRP3, and AL135999.1 expression did not favor the PSA-free survival time, whereas AL360181.4 did (Supplemental Fig. 2B).

### Validation of the risk model

**Fig. 4:**
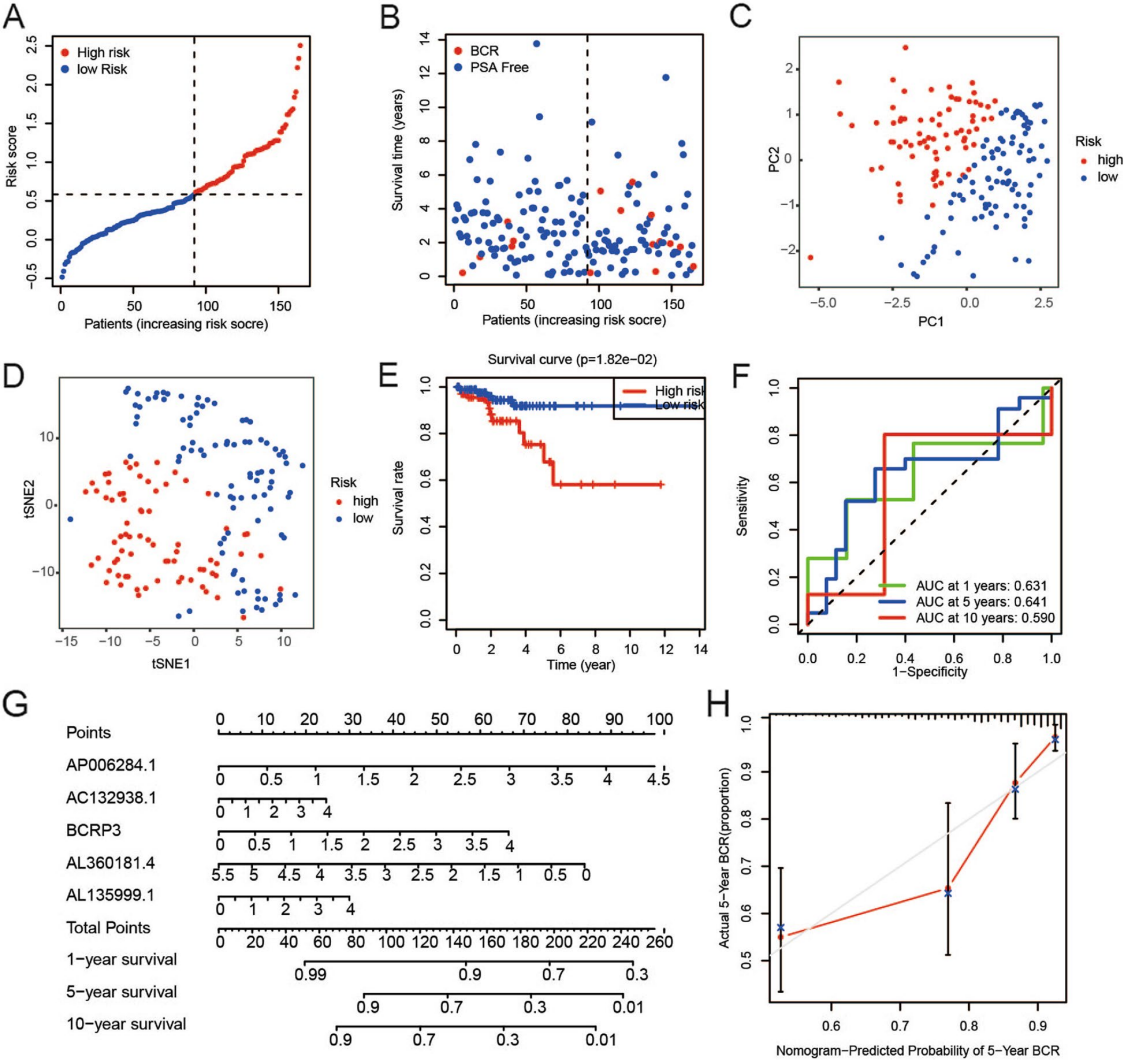
Validation of the prognostic model in the verification group. **A**-**B** The scatter plots showed that different risk scores indicated different BCR states of PCa patients in the verification group. **C**-**D** Effective clustering ability of risk score based on the 5 frlncRNAs in the verification group was shown. **E** There was a distinct difference in PSA-free survival time between high- and low-risk groups in the verification cohort. **F** ROC showed the predictive value of risk score for BCR in the verification cohort. **G** The nomogram was constructed based on the 5 frlncRNAs. **H** The calibration plot for the prediction of nomogram at 5 years

To determine the reliability and stability of the prediction model, it was used to validate data in the verification cohort. Individuals in the verification cohort were divided into high- and low-risk groups in the manner described above. The risk scores and BCR states of patients were visualized (Fig. 4A-B). Findings from PCA and t-SNE analysis validated that the samples in the high- and low-risk groups were distributed separately (Fig. 4C-D**)**. KM plots showed that the PSA-free survival of patients with PCa in the low-risk cohort was markedly better than that in the high-risk cohort (Fig. 4E). ROC curve analysis showed that this model had good predictive efficiency (AUC = 0.631 for 1 year, 0.641 for 5 years, and 0.590 for 10 years) (Fig. 4F). A model was developed by incorporating the five frlncRNAs and presented as a nomogram (Fig. 4G). Each factor was assigned a specific weighted score in the nomogram. The total score was calculated by adding the points for each frlncRNA. The calibration plot of the nomogram showed high consistency between the predicted and actual probabilities of 5-year PSA-free survival, indicating its superiority in clinical practice (Fig. 4H).

The risk scores and BCR states were visualized for all patients with PCa as well (Supplemental Fig. 3A-B). PCA and t-SNE analysis were also conducted (Supplemental Fig. 3C-D). Patients with high-risk scores showed a shorter PSA-free time (Supplemental Fig. 3E). ROCs for all patients generated with AUCs at 1 year, 5 years, and 10 years were 0.776, 0.755, and 0.680, respectively (Supplemental Fig. 3F).

### Association of risk scores and clinical characteristics

**Fig. 5:**
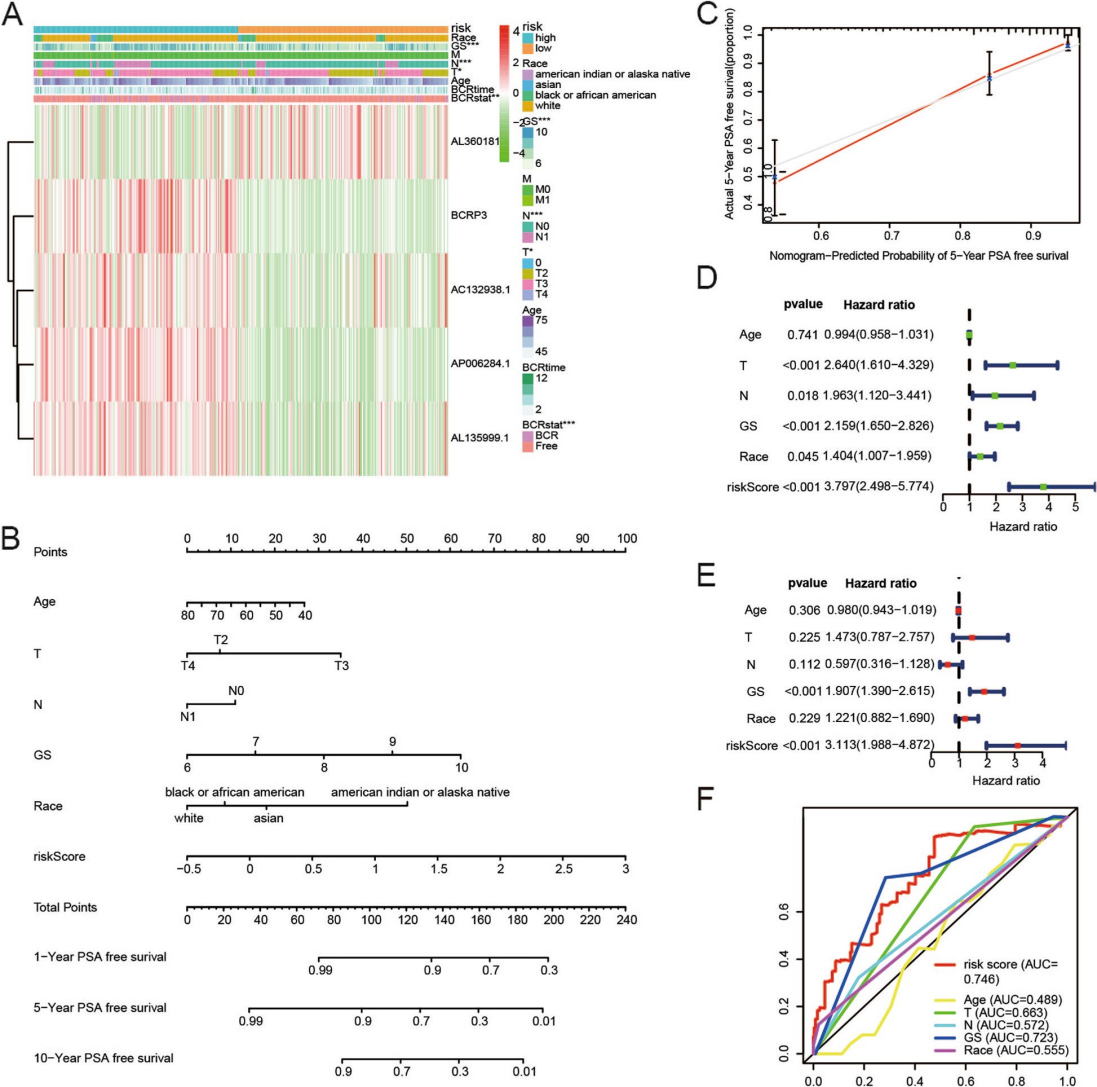
Correlation between risk scores and clinical features. **A** The relationships between risk score and clinicopathological features were investigated using Chi-square test. **B**-**C** The nomogram integrated with age, T stage, N stage, Gleason score, race and risk score and the calibration plot were constructed. **D**-**E** Univariate and multivariate Cox regression model confirmed that risk score was an independent prognostic predictor of BCR. **F** ROCs of indexes for predicting BCR of PCa

The heatmap displayed the correlation between the expression of the five frlncRNAs and the clinical traits in the two groups. The risk groups were found to be strongly associated with the Gleason score, N stage, T stage and BCR states whereas they were not associated with race, M stage, age and BCR time (Fig. 5A).

Likewise, a model that incorporated the risk score and clinicopathological parameters was developed and illustrated as a nomogram to predict the 1-, 5-, and 10-year PSA-free survival probability rates (Fig. 5B). A calibration plot was also generated (Fig. 5C). To confirm whether the risk score was an independent prognostic factor, univariate and multivariable Cox regression analyses were performed. The results of the univariate Cox regression analysis indicated that T stage, Gleason score, and risk score were factors for predicting poor survival (Fig. 5D). Multivariate Cox regression analysis showed that only the risk score was an independent prognostic factor (Fig. 5E). ROCs showed that the risk score had the highest AUC at 0.746, which was greater than that of other clinical factors (age, N stage, T stage, Gleason score, and race) (Fig. 5F).

### Differences in immune cell infiltration between the high- and low-risk groups

**Fig. 6:**
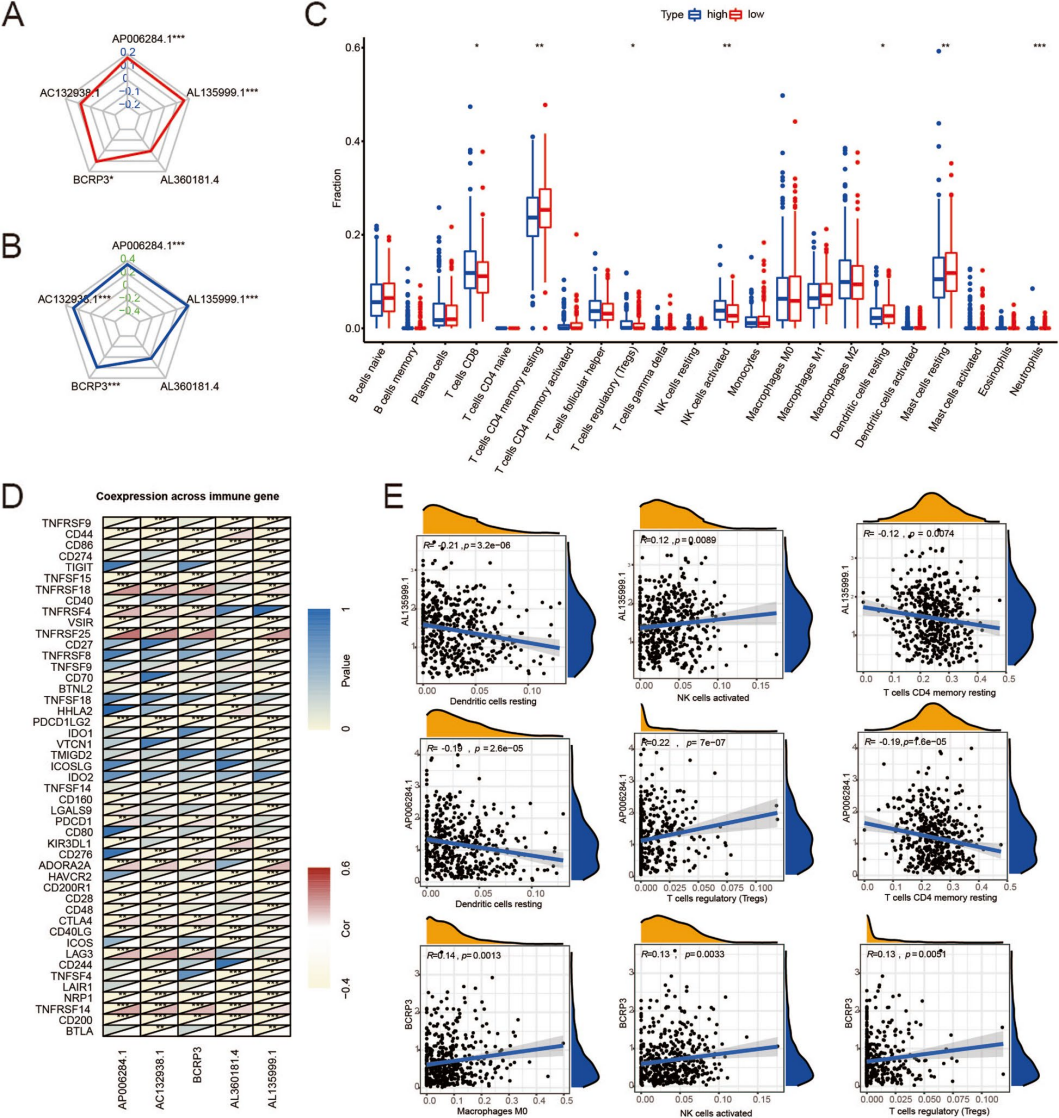
Immune landscape in high- and low-risk groups. **A**-**B** Correlations of frlncRNAs expression with TMB and MSI. **C** Immune cells patterns in different risk groups. **D** The correlation between frlncRNAs and known immune checkpoints. **E** The three immune cells most associated with AP006284.1, BCRP3 and AL35999.1

MSI is caused by functional defects in DNA mismatch repair at the tumor sites and induces a higher frequency of mutation and an increase in TMB. TMB was found to be an effective biomarker for immune checkpoint blockade [19]. The radar plots showed that the expression of AP006284.1, BCRP3, and AL35999.1 was positively correlated with TMB and MSI (Fig. 6A-B). Besides, AC132938.1 expression was found to be positively associated with MSI (Fig. 6B).

We next investigated whether the five lncRNAs were involved immune cell infiltration in PCa. We compared the immune cells of the two groups. The number of CD8 + T cells, CD4 + T cells, resting memory T cells, regulatory T cells (Tregs), activated NK cells, resting dendritic cells, resting mast cells and neutrophils showed statistically significant differences (Fig. 6C). Next, we observed a close correlation between the expression of five frlncRNAs and typical immune checkpoints (Fig. 6D). Figure 6E shows the three immune cells most strongly associated with the expression of AP006284.1, BCRP3, and AL35999.1, implying that AP006284.1, BCRP3, and AL35999.1 are key factors in the efficacy of PCa immunotherapy.

### Enrichment analysis

To identify the molecular mechanisms underlying the differences between the two groups, we conducted enrichment analysis based on data from the low-risk and high-risk groups (Supplemental Fig. 4). In the high-risk group, 72 of 178 gene sets were upregulated, and no gene set among the KEGG gene sets was significantly enriched. In the low-risk group, 106 of 178 gene sets were upregulated, and 42 gene sets among the KEGG gene sets were significantly enriched [20–22].

Based on the normalized enrichment score, the most significantly enriched gene sets in the low-risk group were propanoate metabolism, valine leucine and isoleucine degradation, butanoate metabolism, adherens junction, peroxisome, citrate cycle (TCA cycle), fatty acid metabolism, n-glycan biosynthesis and sphingolipid metabolism, which highlighted the function of metabolic pathways mediated by frlncRNAs in PCa.

### Consensus clustering of ferroptosis-related genes led to the grouping of patients with PCa into three clusters

**Fig. 7:**
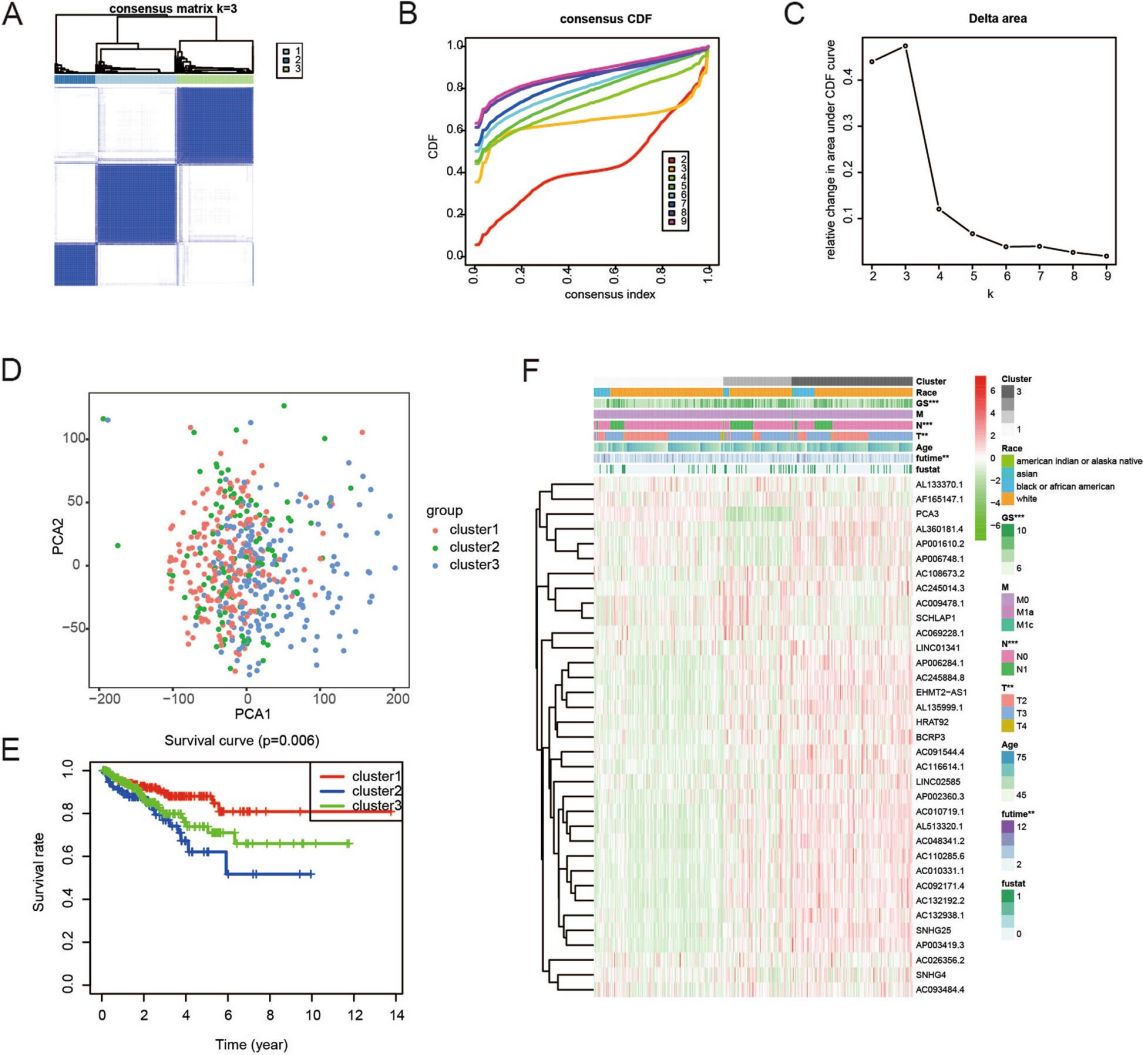
The consensus clustering for 495 PCa samples. **A** Consensus clustering matrix for k = 3. **B** Consensus clustering cumulative distribution function (CDF) for k = 2 to 10. **C** Relative change in area under CDF curve for k = 2 to 10. **D** PCA revealed an effective clustering ability of risk score. **E** KM PSA-free survival curves for PCa patients in the 3 clusters. **F** Heatmap showed clinicopathological features of patients

Based on the expression of the five differentially expressed frlncRNAs, we attempted to classify the 495 PCa samples in TCGA cohort into different subtypes using unsupervised clustering. The clustering variable (k) was increased from 2 to 10. The intragroup correlations were found to be the highest, the intergroup correlations were found to be lower, and no subgroup was too small at k = 3 (Fig. 7A-C). According to the above criteria, the 495 samples were divided into three clusters. The results of PCA indicated that the three clusters were well classified (Fig. 7D). In addition, the PSA-free survival time of cluster 1 was found to be longer than that of cluster 3, which had a lower risk of BCR than cluster 2 (Fig. 7E). Figure 7F shows the clinicopathological characteristics of the three clusters.

### Expression and function of frlncRNAs in PCa

**Fig. 8:**
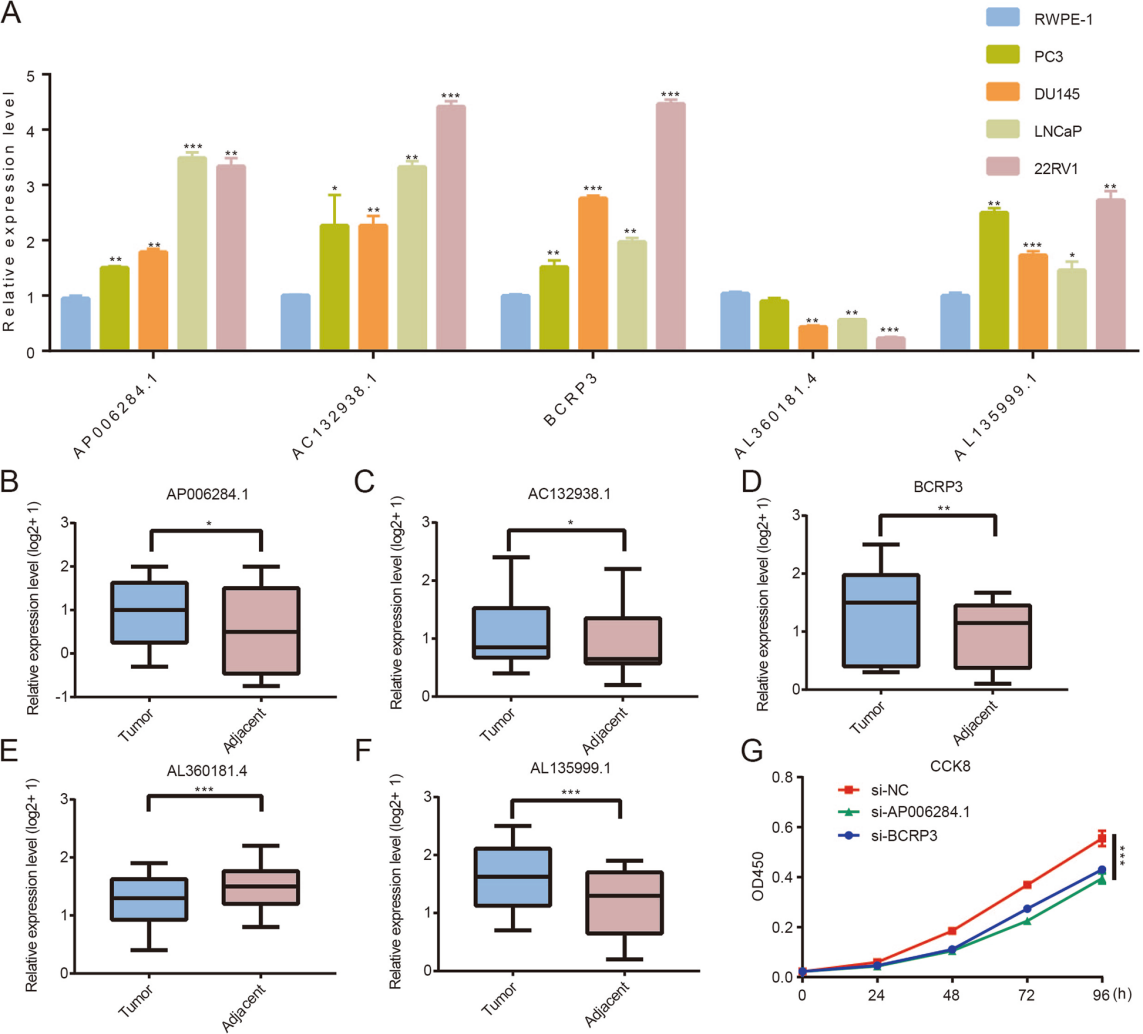
Role of 5 frlncRNAs in PCa. **A** Relative expression of frlncRNAs in PCa cells and RWPE-1 cell. **B**-**F** Relative expression of frlncRNAs in PCa and adjacent tissues. **G** CCK8 assays showed that BCRP3 and AP006284.1 downregulation impaired growth rates in 22RV1 cells

We tested their expression level in the cell lines. As shown in Fig. 8A, compared with that in RWPE-1 cells, AP006284.1, AC132938.1, BCRP3, and AL135999.1 expression was relatively higher in PCa cell lines, but AL360181.4 exhibited the opposite trend. We then validated the expression levels of these five lncRNAs in ten pairs of samples from patients with PCa in our cohort and similar expression trends were observed in clinical samples (Fig. 8B-F). In addition, the findings from the CCK8 assay indicated the downregulation of BCRP3 and AP006284.1 expression inhibited the proliferation of 22RV1 cells (Fig. 8G). Furthermore, BCRP3 knockdown also reduced the expression of RB1, STAT3, and LAMP2 (Supplemental Fig. 5). These results further validated the findings of the bioinformatics analysis.

## Discussion

The BCR of PCa corresponds to a PSA greater than 0.2 ng/mL on two consecutive occasions after radical surgery or radical radiotherapy, along with the absence of recurrent or metastatic lesions in imaging [23]. BCR is a critical factor to evaluate the prognosis of patients with PCa. Therefore, the identification of biological markers for BCR in PCa is vital for improving clinical outcomes. With the rapid development of bioinformatics technology, an increasing number of models have been constructed for the prediction of PCa prognosis [24]. However, most studies have focused on models based on clinical indexes or applied immune-related genes to assess the immune abundance in solid tumors [25]. The models developed by integrating ferroptosis-related genes are emerging as promising strategies to predict the prognosis of male patients with various tumors [26]. Herein, we identified a frlncRNA signature to predict the BCR probability rates and investigated the correlation between immune cell infiltration and the frlncRNA signature.

In this study, patients with PCa in TCGA were randomized into the training and verification cohorts. A total of 35 duplicated lncRNAs were first obtained in both the differential and prognostic analyses. Following this, the five lncRNAs were used to generate the prognostic model by LASSO analysis in the training group. Notably, the accuracy and reliability of the model were validated in the verification group, suggesting that our model was a strong tool for predicting the PSA-free survival time of patients with PCa. Additionally, our results indicated a potential connection between the immune status in the TME and the frlncRNA signature, which could be used as an indicator of the immunotherapy response.

Our results suggested that the five frlncRNAs (AP006284.1, AC132938.1, BCRP3, AL360181.4 and AL135999.1), which could transform the immune landscape, could be used to evaluate the risk of BCR. Some of the lncRNAs were reported to contribute to the occurrence and development of cancer. AP006284.1 served as a prognostic lncRNA in colorectal cancer [27]. Jing Sui et al. concluded that BCRP3 expression correlated with the pathological stage, lymph node metastasis, and overall survival in lung adenocarcinoma [28]. Consistently, our data also supported BCRP3 as an oncogene in PCa. Nonetheless, the mechanisms influencing the expression of the frlncRNAs in PCa remain unknown. In our study, the result of GSEA elucidated that the probable mechanisms involved in the expression of the frlncRNAs in the low-risk group were primarily related to metabolism, a possibility that we will focus on in future studies.

The most important finding of this study was the correlation between frlncRNA expression and immune cell infiltration in PCa. TME is of tremendous significance not only in tumor progression but also in the responses of patients to immunotherapy [29]. Various drugs, such as sorafenib, eliminate tumor cells via ferroptosis [30]. Ferroptosis was reported to promote the suppression of IFNγ expression in response to immune checkpoint blockade, highlighting its crucial function in TME [31]. In this study, we found significant differences in the abundances of CD8 + T cells, CD4 + T cells, resting memory T cells, Tregs, activated NK cells, resting dendritic cells, resting mast cells, and neutrophils between the high- and low-risk groups. Additionally, we also assessed the relationship between frlncRNA expression and known immune checkpoints and observed a strong correlation. Collectively, the findings may help novel strategies for tumor immunotherapy.

GSEA revealed that the expression of the five frlncRNAs was associated with TCA cycle and fatty acid metabolism. Citric acid serves as an important mediator in PCa since citric acid oxidation provides energy for cell proliferation [32]. Consistent with our findings, Liu et al. reported that the instability of IDH1, a key enzyme of TCA cycle, in PCa cells, facilitated glycolysis and subsequent tumorigenesis [33]. Accumulating evidence has confirmed that the dysregulation of fatty acid metabolism plays a vital role in cancer progression. PCa cells have a greater demand for fatty acids, which are used directly in biomass production [34]. The levels of fatty acids correlate with the Gleason score and indicate the aggressiveness of PCa [35]. Overall, our findings support the fact that frlncRNAs regulate PCa development through classical metabolic pathways and also provide a foundation for the identification of drugs that regulate the frlncRNAs levels in cancer cells as therapeutic agents.

To summarize, this study was the first to propose a frlncRNA signature as a clinically adaptable tool for predicting the PSA-free survival time of patients with PCa. We also conducted in vitro experiments to confirm BCRP3 as an oncogene in PCa. Nevertheless, we cannot eliminate the confounding effects of the LASSO regression model based on current data. Additional basic experiments are needed to determine the specific mechanisms of action of frlncRNAs in PCa.

## Supplementary Information

**Additional file 1: Supplemental Figure 1.** Heatmap showed the expression profiles of the 35 lncRNAs in PCa samples and normal controls.

**Additional file 2: Supplemental Figure 2.** Regulatory network and Sankey diagram of the 5 frlncRNAs. (A) The interactive network of frlncRNAs and mRNAs using Cytoscape. (B) Sankey diagram demonstrated the relationship between the 5 frlncRNAs, ferroptosis mRNAs and risk type.

**Additional file 3: Supplemental Figure 3.** The predictive ability of the model in all PCa patients. (A-B) The risk score and BCR states of every individual. (C-D) Effective clustering ability of risk score based on the 5 frlncRNAs in all patients. (E) Patients with high risk scores had shorter PSA-free survival expectancy. (F) ROCs showed the capability of risk score to predict BCR in all patients.

**Additional file 4: Supplemental Figure 4.** The most significantly enriched pathways enriched in the low-risk group [20–22]. (A) propanoate metabolism. (B) valine leucine and isoleucine degradation. (C) butanoate metabolism. (D) adherens junction. (E) peroxisome. (F) citrate cycle tca cycle. (G) fatty acid metabolism. (H) n glycan biosynthesis. (I) sphingolipid metabolism. All FDR<0.25 and *P*<0.05.

**Additional file 5: Supplemental Figure 5.** Downstream of BCRP3. (A-B) Verification of knockdown efficiency. (C) Effects of BCRP3 on RB1, STAT3 and LAMP2 mRNA levels.

**Additional file 6: Supplemental Table 1.** The PCR primers and siRNAs sequences in this study.

**Additional file 7: Supplemental Table 2.** A total of 669 frlncRNAs were identified by Pearson’s correlation analysis in 495 prostate patients with absolute correlation coefficient >0.30 and *P* < 0.001.

**Additional file 8: Supplemental Table 3.** Univariate Cox analysis was conducted and 183 lncRNAs were verified to be associated with BCR.

**Additional file 9: Supplemental Table 4**. Baseline characteristics of enrolled cases.

## Data Availability

All of the date used in this study can be downloaded from UCSC Xena (https://xenabrowser.net/datapages/): cohort:TCGA Prostate Cancer (PRAD) and GENCODE databank (https://www.gencodegenes.org/): Long non-coding RNA gene annotation as our described in the methods.
