# Supplementary figures and images for: The ferroptosis-related long non-coding RNAs signature predicts biochemical recurrence and immune cell infiltration in prostate cancer
Source: BMC Cancer. 2022 Jul 18;22:788. doi: 10.1186/s12885-022-09876-8 (PMC9290257; doi:10.1186/s12885-022-09876-8)

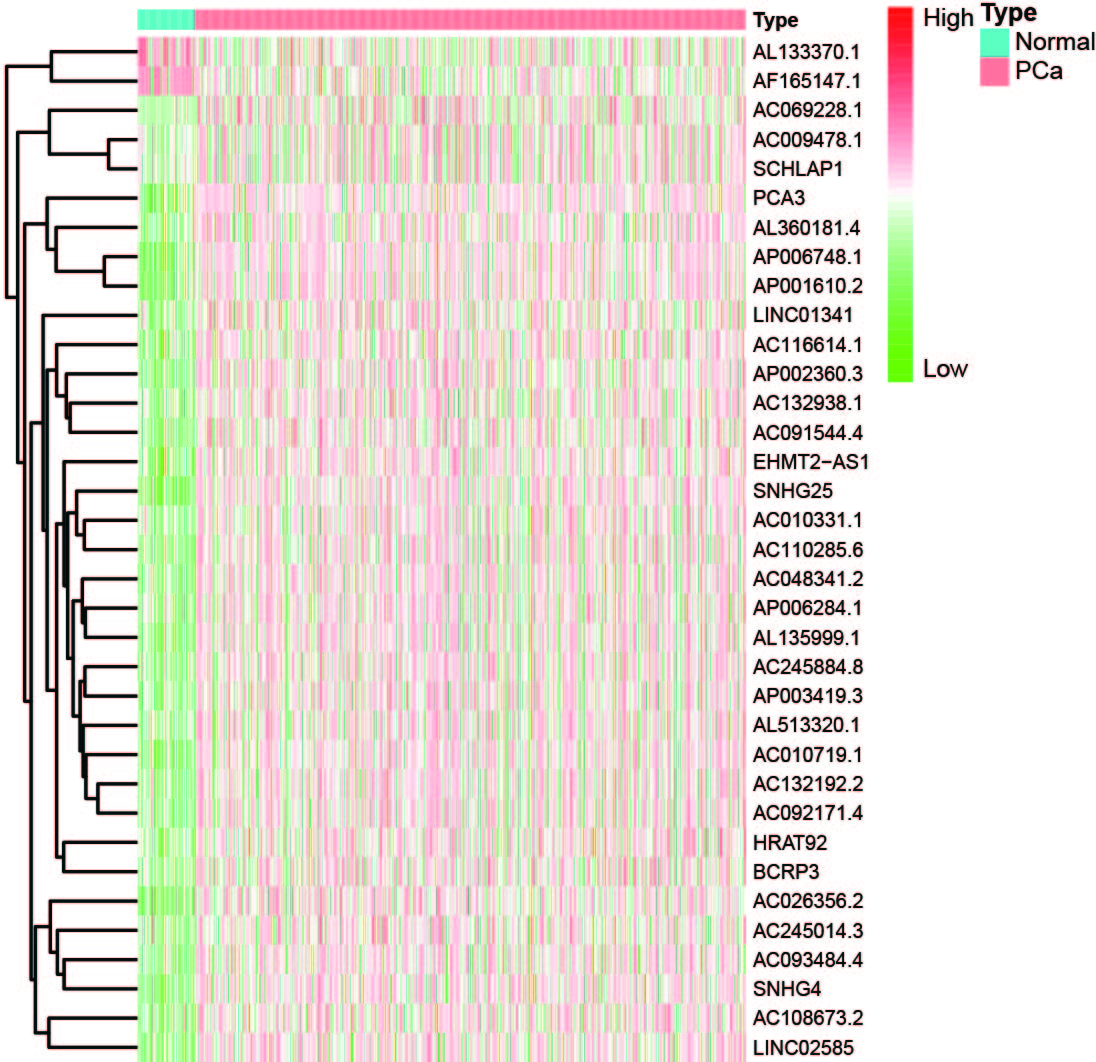

Supplement: Supplementary file 1 — Additional file 1: Supplemental Figure 1. Heatmap showed the expression profiles of the 35 lncRNAs in PCa samples and normal controls. [file 12885_2022_9876_MOESM1_ESM.jpg]

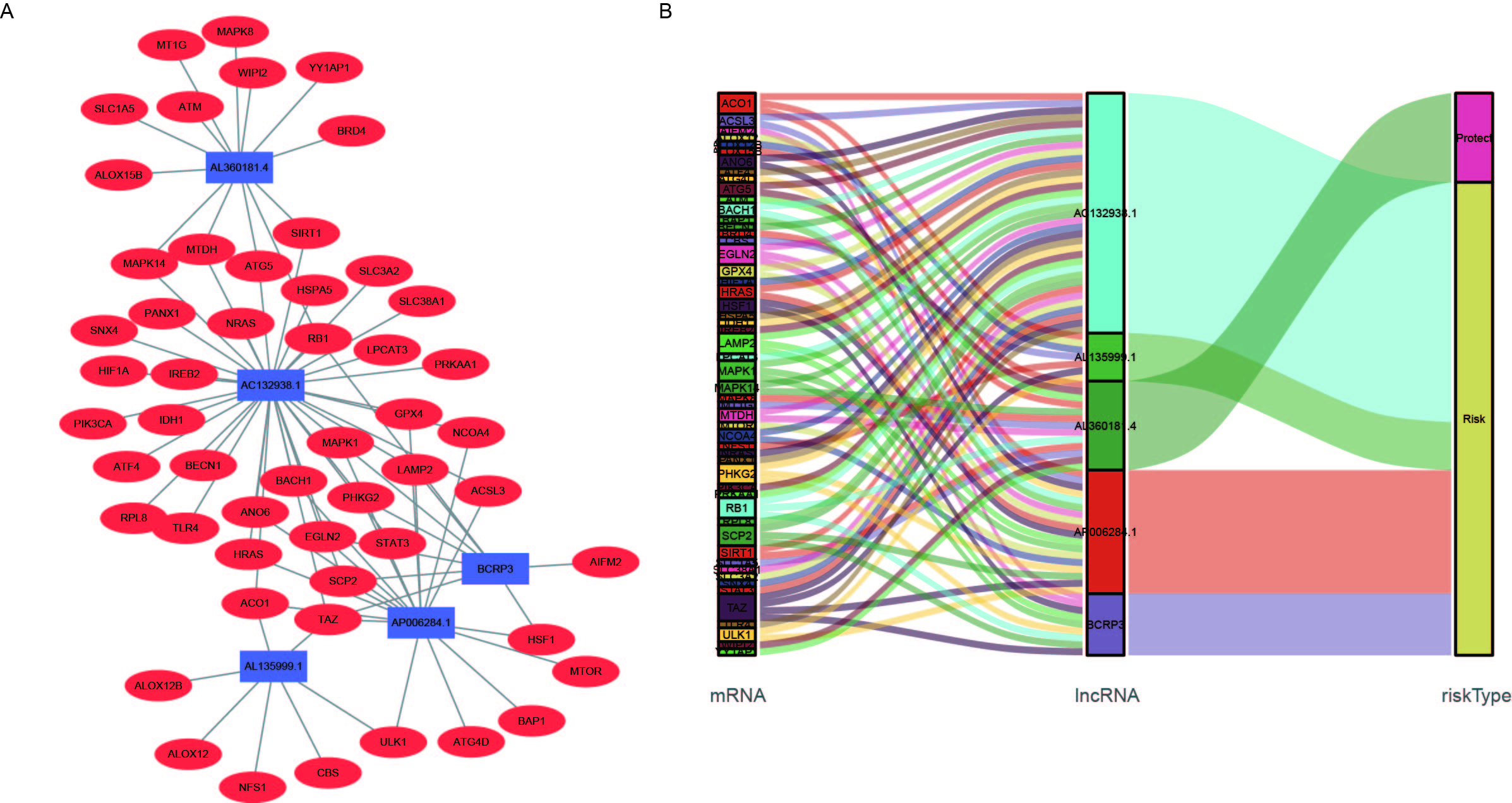

Supplement: Supplementary file 2 — Additional file 2: Supplemental Figure 2. Regulatory network and Sankey diagram of the 5 frlncRNAs. (A) The interactive network of frlncRNAs and mRNAs using Cytoscape. (B) Sankey diagram demonstrated the relationship between the 5 frlncRNAs, ferroptosis mRNAs and risk type. [file 12885_2022_9876_MOESM2_ESM.jpg]

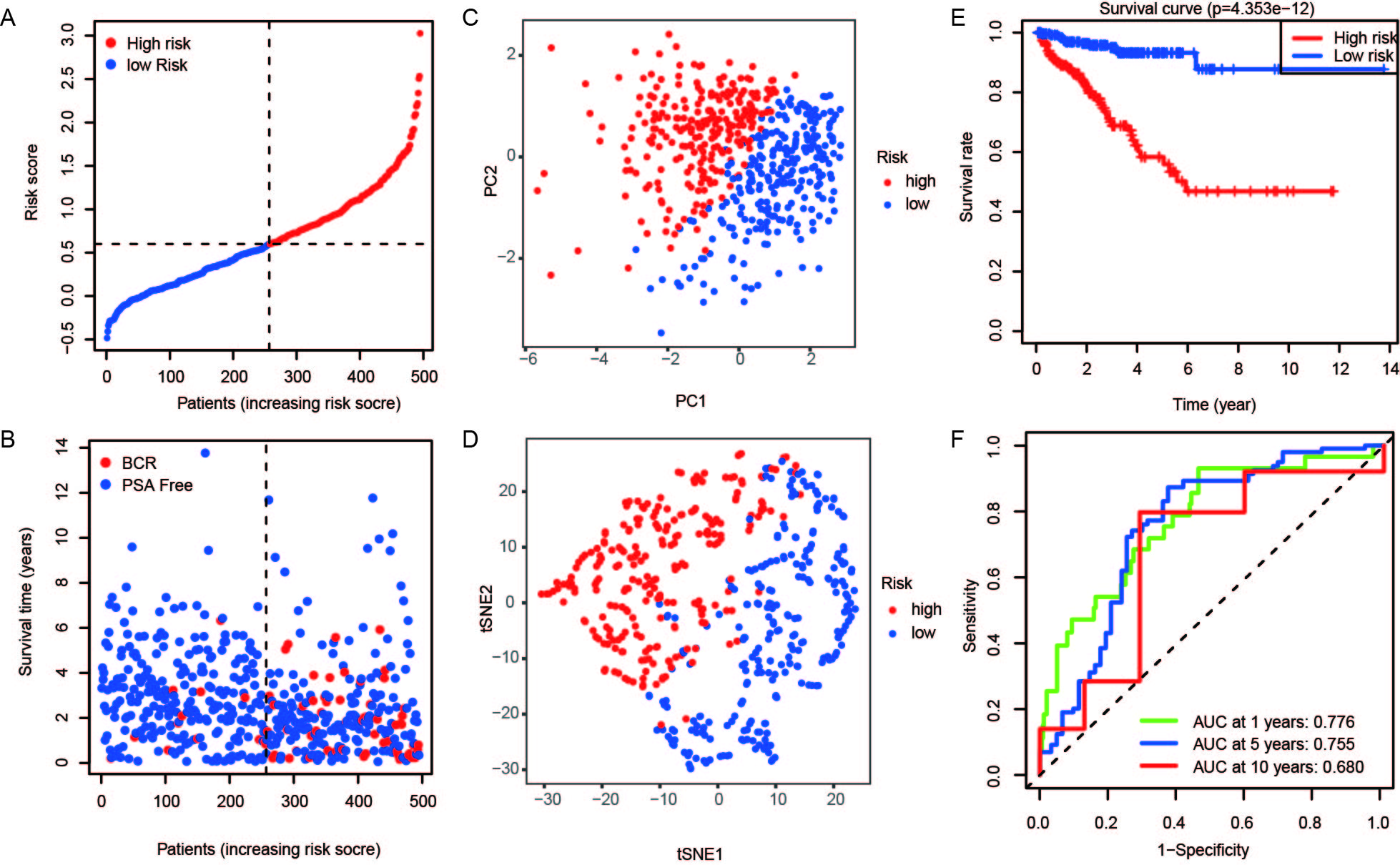

Supplement: Supplementary file 3 — Additional file 3: Supplemental Figure 3. The predictive ability of the model in all PCa patients. (A-B) The risk score and BCR states of every individual. (C-D) Effective clustering ability of risk score based on the 5 frlncRNAs in all patients. (E) Patients with high risk scores had shorter PSA-free survival expectancy. (F) ROCs showed the capability of risk score to predict BCR in all patients. [file 12885_2022_9876_MOESM3_ESM.jpg]

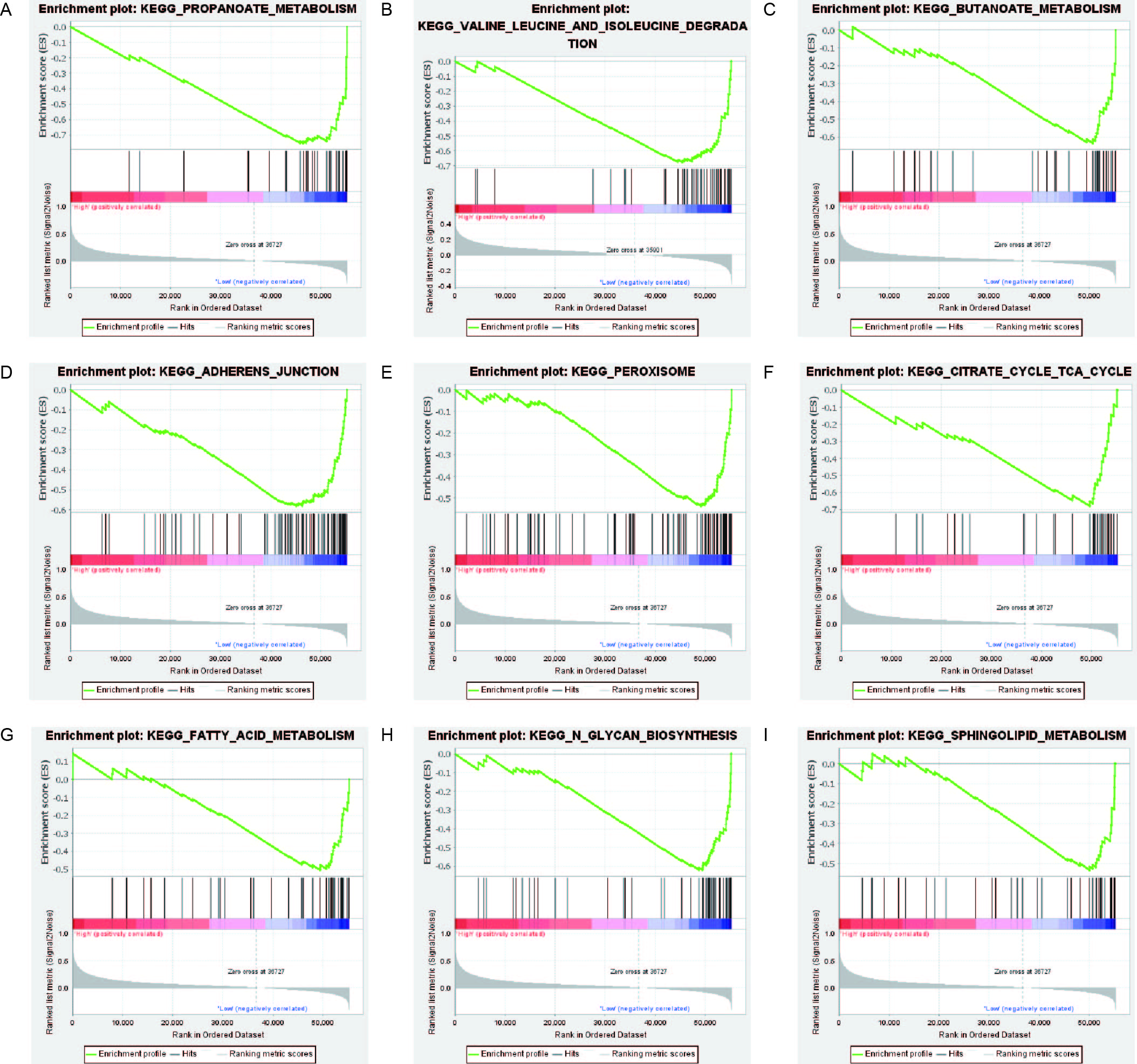

Supplement: Supplementary file 4 — Additional file 4: Supplemental Figure 4. The most significantly enriched pathways enriched in the low-risk group [20–22]. (A) propanoate metabolism. (B) valine leucine and isoleucine degradation. (C) butanoate metabolism. (D) adherens junction. (E) peroxisome. (F) citrate cycle tca cycle. (G) fatty acid metabolism. (H) n glycan biosynthesis. (I) sphingolipid metabolism. All FDR<0.25 and P<0.05. [file 12885_2022_9876_MOESM4_ESM.jpg]

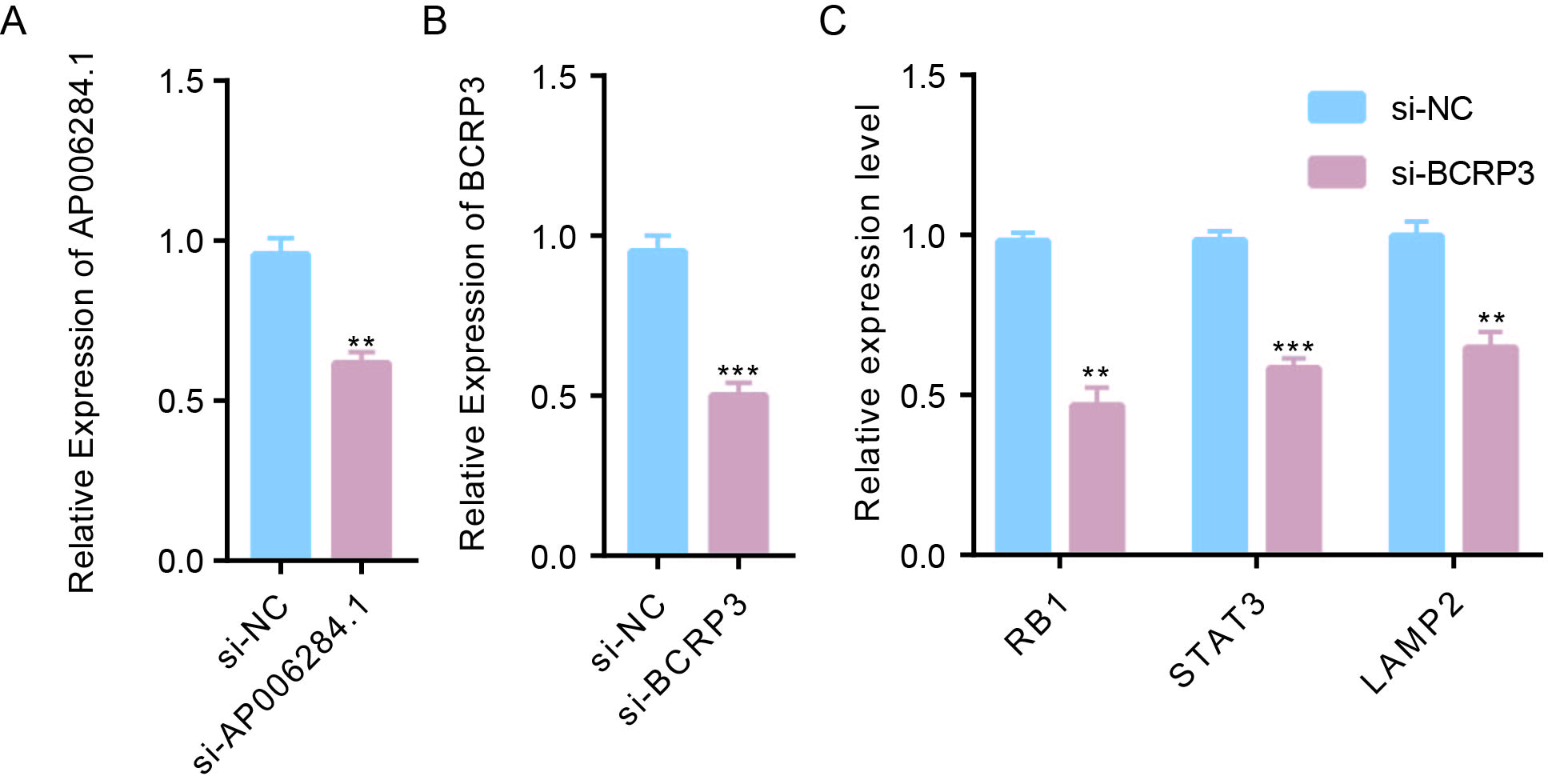

Supplement: Supplementary file 5 — Additional file 5: Supplemental Figure 5. Downstream of BCRP3. (A-B) Verification of knockdown efficiency. (C) Effects of BCRP3 on RB1, STAT3 and LAMP2 mRNA levels. [file 12885_2022_9876_MOESM5_ESM.jpg]
