# Supplementary material for: The ferroptosis-related long non-coding RNAs signature predicts biochemical recurrence and immune cell infiltration in prostate cancer
Source: BMC Cancer. 2022 Jul 18;22:788. doi: 10.1186/s12885-022-09876-8 (PMC9290257; doi:10.1186/s12885-022-09876-8)
Supplement: Supplementary file 6 — Additional file 6: Supplemental Table 1. The PCR primers and siRNAs sequences in this study. [file 12885_2022_9876_MOESM6_ESM.docx]

**Supplemental Table 1** The PCR primers and siRNAs sequences in this study.

Primers 5’-3’ sequence

AC132938.1-forward TCTGACGGTGCCTGTGGGTGT

AC132938.1-reverse TTGTGCGGTGCATCTGGAGTT

AL135999.1-forward GAGCAGTGGGAGGAAAAGGAGA

AL135999.1-reverse CTGTGGGTGAAGAAAGAGTGAA

AL360181.4-forward AGCCGCCAGACAGAATGAGGA

AL360181.4-reverse ATTACAGGAATGAACCACCGC

AP006284.1-forward AGCGATGGGACAGGACAAGACT

AP006284.1-reverse CCAAGACACCAAGAGCTGAGAA

BCRP3-forward TATTGGACGCTGGACCCGCA

BCRP3-reverse CTTGACTCCGAAGACCCCTGT

LAMP2-forward CTGGAGTAGAAAGTTAGATTGC

LAMP2-reverse AGCCACTGTTTTTTATGATGAC

RB1-forward CTCCTAAACCACTGAAAAAACT

RB1-reverse ATCTGTGAGAGACAATGAATCC

STAT3-forward GGGTGCTTACAACCTTGACTCC

STAT3-reverse ACTCTCTCCCCCTCTTCTTCCA

siRNAs

si- AP006284.1 GGGCAUUUCUGGUUAGAAATT

si- BCRP3 UUUAAUGAAAGUGUCGGCCTT

Negative control UUCUCCGAACGUGUAACGUTT
