# Supplementary material for: The ferroptosis-related long non-coding RNAs signature predicts biochemical recurrence and immune cell infiltration in prostate cancer
Source: BMC Cancer. 2022 Jul 18;22:788. doi: 10.1186/s12885-022-09876-8 (PMC9290257; doi:10.1186/s12885-022-09876-8)
Supplement: Supplementary file 9 — Additional file 9: Supplemental Table 4. Baseline characteristics of enrolled cases. [file 12885_2022_9876_MOESM9_ESM.docx]

Supplementary Table 4 Baseline characteristics of enrolled cases.

Characteristics TCGA cohort n=495 No. of patients (%)

Status

BCR 71 (14.34)

Non-BCR 424 (85.66)

Age

≤50 35 (7.07)

51~60 184 (37.17)

61~70 231 (46.67)

>70 35 (7.07)

Unknown 10 (2.02)

Pathological_T

0 1 (0.20)

T2 187 (37.78)

T3 291 (58.79)

T4 10 (2.02)

Unknown 6 (1.21)

Pathological_N

N0 344 (69.49)

N1 78 (15.76)

Unknown 73 (14.75)

Pathological_M

M0 453 (91.52)

M1 3 (0.61)

Unknown 39 (7.88)

Race

American indian or alaska native 1 (0.20)

Asian 12 (2.42)

Black or African American 56 (11.31)

White 412 (83.23)

Unknown 14 (2.82)

Follow-up time

≤365 78 (15.76)

366~1825 332 (67.07)

>1825 85 (17.17)
